# Receiver–Coil Location Detection in a Dynamic Wireless Power Transfer System for Electric Vehicle Charging

**DOI:** 10.3390/s22062317

**Published:** 2022-03-17

**Authors:** Mattia Simonazzi, Leonardo Sandrolini, Andrea Mariscotti

**Affiliations:** 1 Department of Electrical, Electronic and Information Engineering (DEI), University of Bologna, 40136 Bologna, Italy; mattia.simonazzi2@unibo.it (M.S.); leonardo.sandrolini@unibo.it (L.S.); 2 Department of Electrical, Electronics and Telecommunication Engineering and Naval Architecture (DITEN), University of Genova, 16145 Genova, Italy

**Keywords:** wireless power transfer, WPT, resonator array, position sensing, circuit modeling, resonant circuits

## Abstract

Receiver position sensing is investigated in a dynamic wireless power transfer (DWPT) system for electric vehicle (EV) charging. Exploiting the peculiar behaviour of the resonator arrays input impedance, it is possible to identify the position of the receiver coil by exciting the first array resonator with a signal at a proper frequency and measuring the resulting current. An analytical expression of the input impedance of the resonator array coupled with the EV receiver coil placed in a generic position is provided; its sensitivity to different circuit parameters is also analysed. The outline of a simple and effective algorithm for the localization of the EV is proposed and applied to a test case.

## 1. Introduction

Wireless Power transfer (WPT) is a promising contactless technology applied to transfer power from a transmitter to a receiver with the purpose of providing supply or charging the receiver battery system. The most diffused solution is that of inductive power transfer (IPT), especially for medium- to high-power applications, or for embedded receivers (such as in the case of implantable devices). One of the most popular applications is that of the recharging of electric vehicles (EVs) of various sizes, from cars to buses and people movers [1,2,3,4]. For this application, we may further distinguish static and dynamic charging, with the receiving vehicle in parked or moving conditions, respectively. In both cases, correct positioning and minimal misalignment are paramount to improve the efficiency that depends on the established mutual coupling between the charging system and the vehicle on-board coil [5,6].

In addition, in the case of dynamic charging, with the transmitter coils being located slightly beneath the road/track level (the primary pads or resonators), the charging system is energized upon the vehicle arrival for a matter of economy, but also protection of pedestrians. In fact, the presence of the vehicle body prevents direct occupation of the pad area and partially shields the the emitted magnetic field [7,8].

In dynamic IPT scenarios, the moving vehicle has a short time available for recharging, proportional to the length of the coupling area and inversely proportional to its speed. To increase the coupling area length, multiple charging coils may be used. Without correspondingly multiplying the complexity and cost of the charging lane, an array of cascaded resonating coils may be used, where the transmitter directly feeds the first coil and the magnetically coupled vehicle resonator, while passing over the array, takes power flowing from the same unique transmitter through the intermediate relay coils [9,10].

In an IPT highway perspective with primary pads located one after the other, EV position detection may be exploited to energize the primary pad(s) containing the vertical projection of the EV on-board coil, providing the best coupling. Speed of travel may be in the range of 50 to 100 km/h, corresponding to 27.8 to 56.6 m/s. With primary pads of a length slightly longer than the EV coil (approximately 1 m to fix ideas), complete EV passing over each pad requires 17 to 34 ms and coil switching operation including detection should take about 1 ms or so. Considering instead a static IPT charging scenario, accurate alignment with the IPT pad at ground may be challenging, as the longitudinal misalignment resulting from various types of dynamic tests was consistently on average about 70 cm, with a standard deviation of about 50 cm or larger [11]. Longitudinal errors seem to be much more relevant than lateral displacement that was observed in the order of 10 cm. The presence of rear and side barriers (hard steel structure, soft bumper, other cars), as well as the standard parking sensor equipment of the participating vehicles, had a significant influence on the achieved accuracy.

A solution for EV detection and correction of misalignment errors is thus relevant for the overall efficiency and functionality of IPT (and WPT in general) in static and dynamic conditions. EV detection, combined with position or misalignment measurement, can be achieved by various techniques [12]:

- completely separate sensors, based on other techniques such as optical (video camera) [13], tag readers (RFID technology) [14], GPS [15], etc.;
- additional inductive sensors embedded with the IPT coils, such as interposed detection coils [16,17], Hall sensor array [12], ferrite antenna [18] or TMR (tunneling magnetoresistive) sensors foil [19];
- special design of transmitter and receiver coils, such as overlapping D- and Q-coils, as described in [20];
- exploiting existing IPT coils of a resonating array, determining the behavior of the circuit as a function of position.

Besides the general robustness and accuracy of magnetic coupling methods, highlighted in [12], those exploiting IPT coils are non invasive and do not require substantial modifications to the transmitting and receiving circuits. Operation during the charging process is possible if the test signal uses a non-overlapping band well above the standardized IPT operating frequency intervals, not to be disturbed by the inverter main emissions. The IPT system is modeled and probed at the accessible transmitter port for its electrical characteristics (in the present case input impedance), as a function of receiver position.

Section 2 considers the array of resonant coils and its electrical behavior, in order to derive an expression for the input impedance at the test frequency range. It will be explained in Section 3 that a dissipative termination operating at the test frequency range on the last coil is beneficial to limit the variation of the input impedance and improve detection. Section 4 then demonstrates the algorithm for the interpretation of input impedance values and determination of the receiver coil position.

## 2. Resonator Array Equivalent Circuit

**Figure 1:**
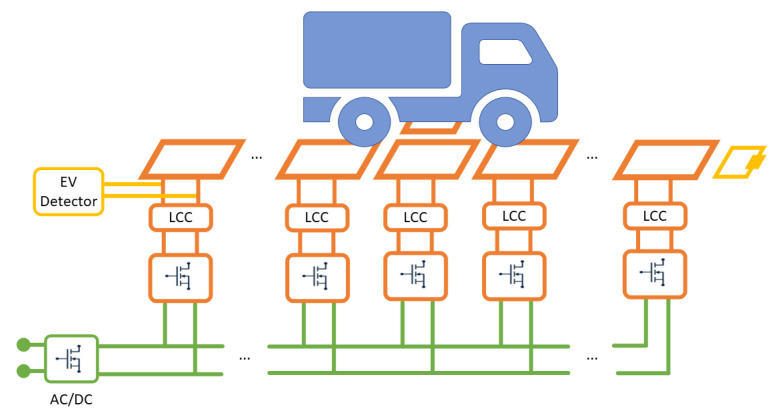
Schematic of a dynamic IPT system. The EV Detector block implements the proposed algorithm and carries out the impedance measurement at the coil terminals.

Dynamic wireless power transfer systems for roadways or [2,21,22] industrial applications [23] are basically composed of an array of coils disposed along the movement direction of the receiver coil, each of them fed by a proper supply system, as depicted in Figure 1. The basic idea that is generally followed consists of turning on only the coil facing the receiver, while the others are kept off. As a result, for each receiver position, the system operates as a simple two-coil IPT apparatus and the dynamic charging is possible with the timely trigger of the coil facing the moving receiver. The coil drivers are assumed to operate at $f_{0}$ = 85 kHz, as suggested in the automotive standard regulating the static WPT systems SAE J2954 [24].

**Figure 2:**
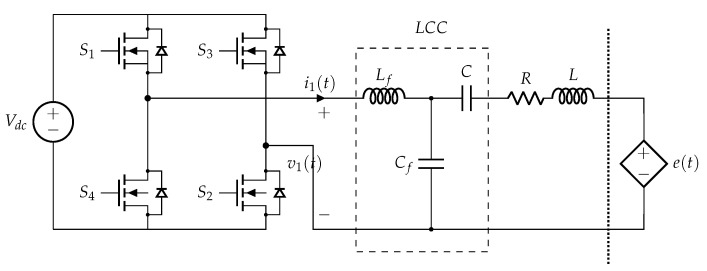
Transmitting coil equivalent circuit, composed of an H-bridge inverter, LCC compensation network and winding model with a series controlled voltage source to represent the coupling with the receiver and adjacent coils.

In particular, each transmitting coil is fed by an H-bridge inverter trough an LCC compensation network, as illustrated in Figure 2. The LCC network has been chosen since, in the case of double sided compensation, it guarantees that the resonant frequency is independent on the coupling coefficient, which strongly varies in dynamic WPT systems, and also independent on the load condition. These peculiarities allow the system to operate at a constant switching frequency [25]. The tuning of the primary compensation network is realized such that:

(1) $$\omega_{0}L_{f}=\frac{1}{\omega_{0}C_{f}}\;,\;\omega_{0}L-\omega_{0}L_{f}=\frac{1}{\omega_{0}C}$$

where $L_{f}$, $C_{f}$ and *C* are the parameters of the lumped components forming the LCC compensation network and *L* the self-inductance of the transmitting coils. In particular, $L_{f}$ is chosen to ensure ZVS operations of the inverter. While the WPT operations are the typical ones of classical dynamic WPT systems, the behaviour when considering the coupling between adjacent coils deserves to be analysed. In this case, the key parameter is the impedance seen by the controlled voltage source e(t), which represents the voltage induced by the adjacent windings.

**Figure 3:**
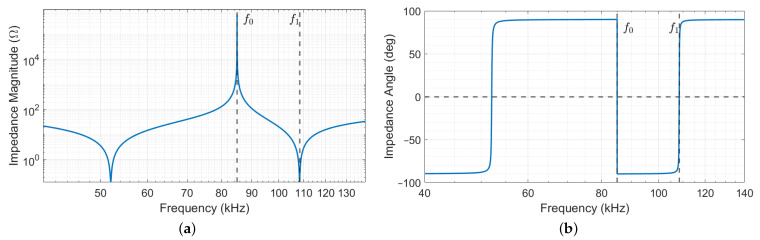
(**a**) Magnitude and (**b**) phase of the driver coil impedance $\hat{Z}$ as a function of the frequency.

The inverter can be assumed to operate as an ideal voltage source, as demonstrated in [5], where its output impedance was shown to be equal to $\frac{8}{\pi^{2}R_{on}}$ that, in the present case, is less than a few mΩ. Such output impedance is more than an order of magnitude smaller than the coil resistance:

(2) $$\hat{Z}\left(\omega \right)=R+j\omega L+\frac{1}{j\omega C}+\frac{j\omega L_{f}\frac{1}{j\omega C_{f}}}{j\omega L_{f}+\frac{1}{j\omega C_{f}}},$$

which is plotted in magnitude and phase as a function of the frequency in Figure 3a,b, respectively. The used parameters’ values are reported in Table 1. The amplitude curve in Figure 3a shows two anti-resonance peaks, below ( 52 kHz) and above ( 108.4 kHz) the central 85 kHz WPT operating frequency; the rest of the discussion is then focused on the second anti-resonance without a loss of generality.

Figure 3b clearly indicates that resonance occurs at two different frequencies, namely $\omega_{0}$ and $\omega_{1}>\omega_{0}$. However, the behaviour of $\hat{Z}$ at the two resonant frequencies is completely opposite, its magnitude maximum being at $\omega_{0}$ and minimum at $\omega_{1}$ (properly said anti-resonance and resonance, but for brevity indicated as resonances when no misunderstanding can occur), as it is possible to see from Figure 3a. This means that, in case of voltage induced at the operating frequency $\omega_{0}$, no current circulates in the coil, which behaves as an open circuit. Reversely, the current is emphasized if the coil is excited at the frequency $\omega_{1}$, as it happens in series-resonant coils. These considerations hold whether the inverter is working or not, its impedance being negligible with respect to $j\omega L_{f}$ due to the MOS body diodes (see Figure 2), which always allow the current to flow back to the DC-bus limited by their internal resistance only.

For what concerns the receiver, the equivalent impedance seen by the correspondent controlled voltage source presents one single resonant frequency at $\omega_{0}$, provided an LCC, series or parallel, compensation network is chosen [6,25,26]. This is due to the equivalent load of the on-board charger, which can be modelled depending on the particular circuit topology as described in [27,28].

As a result, at the angular frequency $\omega_{1}$, the system behaves as an array of magnetically coupled resonators of the type described in [5], with a non-resonant receiver over it.

**Figure 4:**
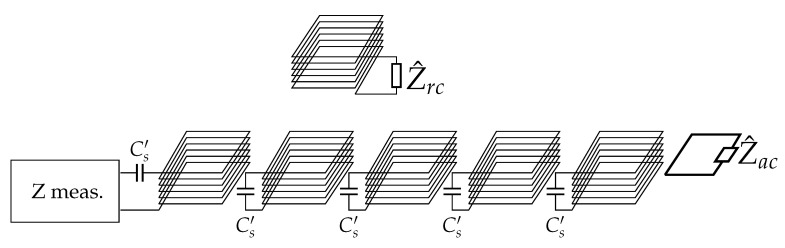
High-frequency equivalent circuit of the resonator array at $\omega_{1}$ with an additional termination coil (with ${\hat{Z}}_{acT}$) and the receiver coil terminated on the impedance ${\hat{Z}}_{rc}$. The “Z measurement” block estimates the array input impedance as described in Section 4.3.

**Figure 5:**
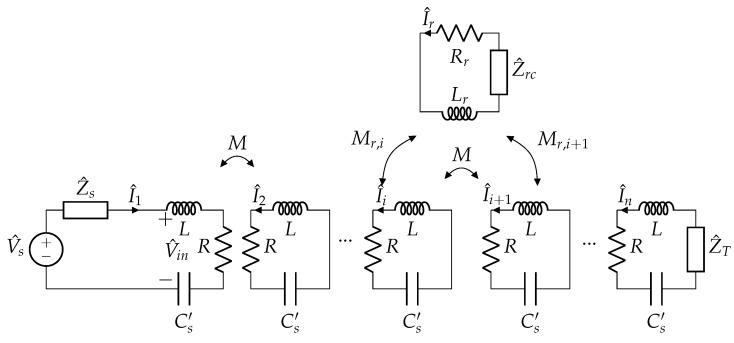
Equivalent circuit of the resonator array at $\omega_{1}$ with a receiver.

Assuming that all the coils are identical and equally spaced, they can be considered characterized by the same resistance *R*, self-inductance *L* and mutual inductance *M* between adjacent coils. The mutual inductance between nonadjacent coils is neglected, being much smaller than *M*. Exciting the first array coil at $\omega_{1}$, it is possible to model the system as schematically illustrated in Figure 4, where an additional termination coil (that will be discussed later) appears too. The equivalent circuit at the angular frequency $\omega_{1}$ is depicted in Figure 5. An equivalent (and fictitious) series capacitance $C_{s}^{\prime }$ that makes the coil resonate is considered to simplify the illustration, which can be defined as:

(3) $$C_{s}^{\prime }=\frac{1}{\omega_{1}L}.$$

The high-frequency signal generator has been modelled by means of its Thévenin equivalent circuit, comprising an ideal voltage source ${\hat{V}}_{s}$ and an internal impedance ${\hat{Z}}_{s}$ at $\omega_{1}$.

Finally, it must be noticed that, in resonator arrays, a termination impedance ${\hat{Z}}_{T}$ can be added to the last coil, providing a degree of freedom which can modify the behaviour of the system, as discussed in [29,30]. However, in this case, the coils are primarily devoted to power transfer and thus no additional impedances can be added when operating at $\omega_{0}$. A possible solution is to add a small resonator at the end of the array such that it reflects a certain impedance in the last array coil at $\omega_{1}$, which acts as termination ${\hat{Z}}_{T}$. Thus, the termination impedance ${\hat{Z}}_{T}$ is defined as:

(4) $${\hat{Z}}_{T}=\frac{{\left(\omega_{1}M_{ac}\right)}^{2}}{{\hat{Z}}_{ac}}$$

where $M_{ac}$ is the mutual inductance between the last array coil and the additional coil and ${\hat{Z}}_{ac}$ its internal impedance, which comprises an arbitrary lumped impedance ${\hat{Z}}_{acT}$ that can be adjusted to achieve the desired ${\hat{Z}}_{T}$. This additional resonator has to be tuned at $\omega_{1}$, so that it does not interfere during charging operations.

## 3. Resonator Array Input Impedance

The position of the receiver can be found measuring the input impedance of the system at $\omega_{1}$, which corresponds to the impedance seen from the first resonator of the array. For practical applications, a high-frequency test signal is applied to the first resonator and propagated along the array.

When deriving the mathematical model in the following, the implicit assumption is that the magnetic coupling between the vehicle receiver and the array coils is subject to vary depending on the vehicle position, but the rapidity of vehicle movement (i.e., its speed) does not cause any appreciable flux variation. In other words, the motional electromagnetic force (emf) is negligible compared to the transformer emf. The total emf can be written by differentiating the flux Φ=Mi, where *M* is the mutual inductance term and *i* is the current flowing in the coupled circuit:

(5) $$\frac{\partial \Phi }{\partial t}=\frac{\partial M}{\partial t}i+M\frac{\partial i}{\partial t}=\frac{\partial M}{\partial x}\frac{\partial x}{\partial t}i+M\frac{\partial i}{\partial t}=\frac{\partial M}{\partial x}vi+M\frac{\partial i}{\partial t}$$

It can be seen that, while overestimating the rate of change of the current *i* considering an operating frequency $f_{1}=\;100\mathrm{kHz}$ and the vehicle speed v≤100 km/h, the motional emf (first term in (5)) is more than two orders of magnitude smaller than the transformer emf (second term in (5)).

### 3.1. Mathematical Model

**Figure 6:**
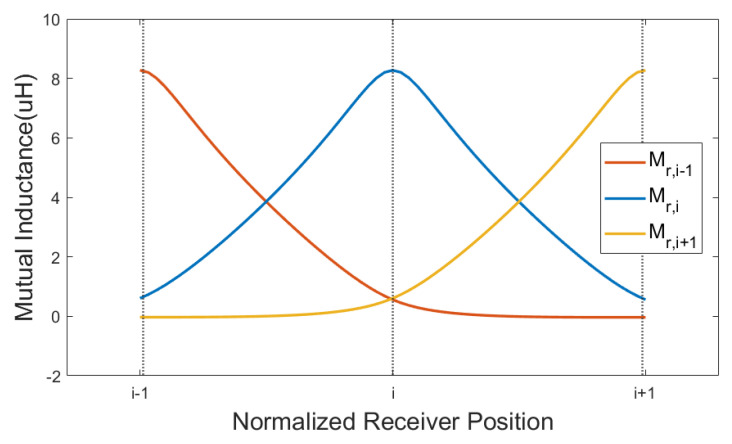
Mutual inductance between the receiver and three consecutive resonators ((i−1)th, *i*th and (i+1)th) of the array, as a continuous function of the position *x*.

Exciting the resonator array with a sinusoidal input voltage at the frequency $f_{1}=\omega_{1}/2\pi$, it is possible to consider all currents and voltages as phasors at the angular frequency $\omega_{1}$. In this conditions, the internal impedance of each array cell is $\hat{Z}=R$, while the receiver impedance is ${\hat{Z}}_{r}=R_{r}+j\omega_{1}L_{r}+{\hat{Z}}_{rc}$, where ${\hat{Z}}_{rc}$ depends on the particular load. The system can be modelled as described in [5], considering that the mutual inductance coefficients between the array coils are all equal and the receiver coupled with both the *i*th and (i+1)th coils. More precisely, the mutual inductance terms $M_{r,i}$ and $M_{r,i+1}$ between the receiver and the facing cells depend on the receiver location, as it is possible to see in Figure 6.

A relative coordinate ξ may be defined as:

(6) ξ=x−(i−1)d

where *x* is the absolute coordinate along which the array lies, *i* indicates the first array cell facing the receiver and *d* is the resonator size in the direction of *x*. The Kirchhoff voltage law (KVL) equations that describe the system can be written as:

(7) $$\begin{array}{cc} -\hat{V_{s}}+{\hat{Z}}_{s}{\hat{I}}_{1}+\hat{Z}{\hat{I}}_{1}+j\omega M{\hat{I}}_{2} & =0\\ j\omega M{\hat{I}}_{1}+\hat{Z}{\hat{I}}_{2}+j\omega M{\hat{I}}_{3} & =0\\ \vdots & \vdots \\ j\omega M{\hat{I}}_{i-2}+\hat{Z}{\hat{I}}_{i-1}+j\omega M{\hat{I}}_{i} & =0\\ j\omega M{\hat{I}}_{i-1}+\hat{Z}{\hat{I}}_{i}+j\omega M{\hat{I}}_{i+1}+j\omega M_{i,r}\left(\xi \right){\hat{I}}_{r} & =0\\ j\omega M{\hat{I}}_{i}+\hat{Z}{\hat{I}}_{i+1}+j\omega M{\hat{I}}_{i+2}+j\omega M_{i+1,r}\left(\xi \right){\hat{I}}_{r} & =0\\ j\omega M{\hat{I}}_{i+1}+\hat{Z}{\hat{I}}_{i+2}+j\omega M{\hat{I}}_{i+3} & =0\\ \vdots & \vdots \\ j\omega M{\hat{I}}_{n-1}+\hat{Z}{\hat{I}}_{n}+{\hat{Z}}_{T}{\hat{I}}_{n} & =0 \end{array}$$

with one additional KVL equation for the receiver:

(8) $$j\omega M_{r,i}\left(\xi \right){\hat{I}}_{i}+j\omega M_{r,i+1}\left(\xi \right){\hat{I}}_{i+1}+{\hat{Z}}_{r}{\hat{I}}_{r}=0$$

In order to write (7) in terms of the array currents only, the receiver coil current ${\hat{I}}_{r}$ obtained from (8) can be substituted into (7). Similarly, a further reduction is also possible substituting in each equation the one relevant to the adjacent resonator, starting from the *n*th one. Then, (7) reduces to the KVL equation of the first resonator:

(9) $$-\hat{V_{s}}+\left[{\hat{Z}}_{s}+\hat{Z}+{\hat{Z}}_{i,i+1}^{eq}\left(\xi \right)\right]{\hat{I}}_{1}=0$$

where ${\hat{Z}}_{i,i+1}^{eq}\left(\xi \right)$ is the equivalent impedance seen from the first cell of the array with the receiver coupled with the *i*th and (i+1)th resonators, which is defined by the recursive formula:

(10) $${\hat{Z}}_{i,i+1}^{eq}\left(\xi \right)=\frac{{\left(\omega M\right)}^{2}}{\hat{Z}\;+\;\frac{{\left(\omega M\right)}^{2}}{\ldots \;+\;\frac{{\left(\omega M\right)}^{2}}{\hat{Z}\;+\;{\hat{Z}}_{d_{i}}\left(\xi \right)\;+\;\frac{{\left(\omega M\right)}^{2}-{\hat{D}}_{i,i+1}\left(\xi \right)}{\hat{Z}\;+\;{\hat{Z}}_{d_{i+1}}\left(\xi \right)\;+\;\frac{{\left(\omega M\right)}^{2}}{\hat{Z}\;+\;\frac{{\left(\omega M\right)}^{2}}{\ldots \;+\;\frac{{\left(\omega M\right)}^{2}}{\hat{Z}\;+\;{\hat{Z}}_{T}}}}}}}}$$

where no implementation,

(11) $${\hat{D}}_{i,i+1}\left(\xi \right)=2j\omega M{\hat{Z}}_{d_{i,i+1}}\left(\xi \right)+{\hat{Z}}_{d_{i,i+1}}^{2}\left(\xi \right)$$

and

(12) $$\begin{array}{cc} {\hat{Z}}_{d_{i}}\left(\xi \right) & =\omega^{2}\frac{M_{i,r}^{2}\left(\xi \right)}{{\hat{Z}}_{r}}\\ {\hat{Z}}_{d_{i+1}}\left(\xi \right) & =\omega^{2}\frac{M_{i+1,r}^{2}\left(\xi \right)}{{\hat{Z}}_{r}}\\ {\hat{Z}}_{d_{i,i+1}}\left(\xi \right)={\hat{Z}}_{d_{i+1,i}}\left(\xi \right) & =\omega^{2}\frac{M_{i,r}\left(\xi \right)M_{i+1,r}\left(\xi \right)}{{\hat{Z}}_{r}}. \end{array}$$

The impedance terms (12) are usually called “defect impedances” and correspond to the reflection impedances of the receiver to the facing array resonators.

In general, for each couple of facing resonators *i* and i+1, the input impedance of the system can be defined as a continuous function of the space. The recursive nature of this formula makes it difficult to express as a function of the absolute coordinate *x*, since the continued fraction changes form according to the receiver position and, to the best of the authors’ knowledge, no closed analytical expressions are known yet for expressions of the type (10). However, they can be easily calculated numerically by means of computers and DSPs and then, in the following, the array input impedance for a generic receiver position *x* is considered and indicated as ${\hat{Z}}_{eq}\left(x\right)$.

### 3.2. Simulations and Discussion

In this paper, an array of six resonators has been considered, whose parameters are reported in Table 1: receiver circuit parameters, which depend on the target application, and the termination impedance that can be adjusted as desired. A typical on-board battery charging system of the type illustrated in [21,24] has been considered as receiver load, which supplies the automotive battery pack described in [31]. The parameters of the receiver circuit are reported in Table 2. While at the resonant frequency $f_{0}$ the receiver impedance ${\hat{Z}}_{r}$ is purely real, when the system is excited at $\omega_{1}$, it presents a capacitive or inductive behaviour depending on the adopted compensation network. In this paper, a series compensation is chosen for the receiver coil, leading to ${\hat{Z}}_{r}=40+j10$ at $\omega_{1}$. The inductive behaviour of this impedance clearly indicates that the receiver does not resonate at $\omega_{1}$.

In general, a resonator array coupled with a receiver can be considered as an extension of the single resonator array, whose input impedance has been deeply investigated in [32]. In particular, for a fixed coil geometry and considering perfect resonance conditions, the input impedance depends on the number of resonators and the termination impedance. In a short-circuited array, the input impedance presents maxima and minima for even and odd numbers of resonators, respectively. The trend is opposite in case of open circuit termination. Moreover, the input impedance is equal to the termination impedance when the array is perfectly matched [30]. Overall, with a real termination impedance still in perfect resonance conditions, the equivalent impedance is real.

**Figure 7:**
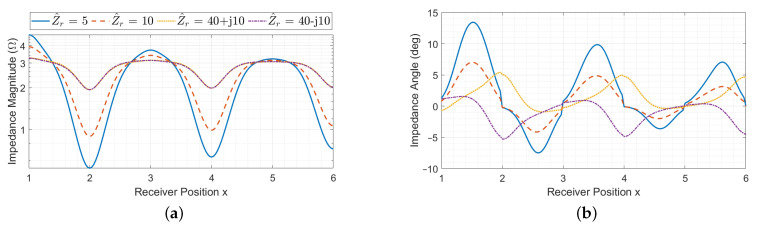
Array input impedance as a function of the receiver position with short-circuit termination for different values of the receiver impedance ${\hat{Z}}_{r}$, in magnitude (**a**) and phase (**b**).

**Figure 8:**
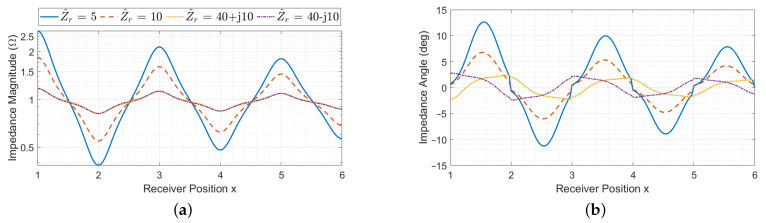
Array input impedance as a function of the receiver position with matched termination for different values of the receiver impedance ${\hat{Z}}_{r}$, in magnitude (**a**) and phase (**b**).

**Figure 9:**
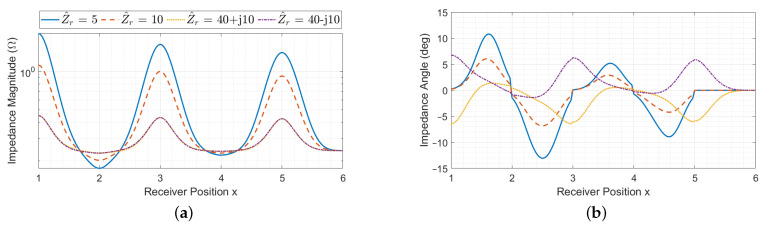
Array input impedance as a function of the receiver position with open-circuit termination for different values of the receiver impedance ${\hat{Z}}_{r}$, in magnitude (**a**) and phase (**b**).

When considering a resonator array with a receiver, its equivalent impedance is also affected by the receiver reflected impedance, whose value can in general be complex and depends on the receiver impedance and position *x*, as it can be seen from (10). Thus, at the cell resonant frequency for a fixed coil geometry, the equivalent impedance depends on the number of resonators, termination impedance, receiver position and receiver impedance. The behaviour of the input impedance ${\hat{Z}}_{eq}\left(x\right)$ is plotted in magnitude and phase in Figure 7, Figure 8 and Figure 9 as a function of the receiver position for different values of receiver impedance ${\hat{Z}}_{r}$ in case of short-circuit, matched and open-circuit terminations of the array, respectively. The values of ${\hat{Z}}_{r}$ have been chosen to represent practical cases.

The input impedance dependency on the receiver parameters is described by the terms (11) and (12). Their effect on the function can be explained considering the system reduced to an array of *i* resonators, terminated with the series of the defect impedance introduced by the receiver and the equivalent impedance ${\hat{Z}}_{n-i,{\hat{Z}}_{T}}^{eq}$ seen from the *i*th cell (corresponding to the input impedance of n−i resonators terminated with ${\hat{Z}}_{T}$) as described in [32,33]. By means of ${\hat{Z}}_{n-\left(i+1\right),{\hat{Z}}_{T}}^{eq}$, it is possible to write ${\hat{Z}}_{i,i+1}^{eq}\left(\xi \right)$ as:

(13) $${\hat{Z}}_{i,i+1}^{eq}\left(\xi \right)=\frac{{\left(\omega M\right)}^{2}}{\hat{Z}\;+\;\frac{{\left(\omega M\right)}^{2}}{\ldots \;+\;\frac{{\left(\omega M\right)}^{2}}{\hat{Z}\;+\;{\hat{Z}}_{d_{i}}\left(\xi \right)\;+\;\frac{{\left(\omega M\right)}^{2}-{\hat{D}}_{i,i+1}\left(\xi \right)}{\hat{Z}\;+\;{\hat{Z}}_{d_{i+1}}\left(\xi \right)\;+\;{\hat{Z}}_{n-\left(i+1\right),{\hat{Z}}_{T}}^{eq}}}}}$$

which clearly indicates that the effect of the receiver can be described by the term:

(14) $${\hat{Z}}_{d_{i}}\left(\xi \right)+\frac{-{\hat{D}}_{i,i+1}\left(\xi \right)}{\hat{Z}\;+\;{\hat{Z}}_{d_{i+1}}\left(\xi \right)\;+\;{\hat{Z}}_{n-\left(i+1\right).{\hat{Z}}_{T}}^{eq}}$$

The presence of maxima and minima in the magnitude and phase of ${\hat{Z}}_{eq}\left(x\right)$ can be led back to the presence of the receiver. In particular, as the magnitude of the receiver impedance becomes smaller, the magnitude of defect impedances increases and they dominate over the equivalent impedance ${\hat{Z}}_{n-i,{\hat{Z}}_{T}}^{eq}$ of the downstream segment of the array (the one after the receiver). Thus, the trend of the input impedance is mainly affected by the number of resonators between the first array cell and the first one covered by the receiver (*i*).

The periodic behaviour of both the magnitude and phase of ${\hat{Z}}_{eq}\left(x\right)$ can be explained considering that ${\hat{D}}_{i,i+1}\left(\xi \right)$, ${\hat{Z}}_{d_{i}}\left(\xi \right)$ and ${\hat{Z}}_{d_{i+1}}\left(\xi \right)$ present the same trend for each pair of resonators *i* and i+1 that face the receiver coil. This can also be confirmed by observing that the behaviour of ${\hat{Z}}_{n-i,{\hat{Z}}_{T}}^{eq}$ is opposite in case of short-circuit and open-circuit terminations, leading to mirrored curves in Figure 7 and Figure 9. Each curve appears to be slightly asymmetric. This peculiarity is due to ${\hat{Z}}_{n-i,{\hat{Z}}_{T}}^{eq}$, which oscillates between very high and low values depending on the number of resonators of the array it is associated with (in this case n−i) and its termination impedance [32]. Although this term (usually real) is negligible with respect to the magnitude of the defect impedances, it slightly affects the denominator of (14).

In case of matched termination, ${\hat{Z}}_{n-\left(i+1\right),{\hat{Z}}_{T}}^{eq}$ behavior is the same for any position and thus the trend of $\varphi_{{\hat{Z}}_{eq}}\left(x\right)$ is symmetric. Furthermore, for increasing values of $|{\hat{Z}}_{r}|$, the defect impedances (12) are smaller and the phase shift of ${\hat{Z}}_{eq}\left(x\right)$ is less pronounced.

Two different situations are discussed: perfectly resonant receiver (meaning that it has a real impedance) and non-resonant receiver. The behaviour of the input impedance ${\hat{Z}}_{eq}\left(x\right)$ is plotted in magnitude and phase in Figure 7, Figure 8 and Figure 9 as a function of the receiver position for different values of receiver impedance ${\hat{Z}}_{r}$ in case of short-circuit, matched and open-circuit terminations of the array, respectively.

#### 3.2.1. Perfectly Resonant Receiver

In this case, the receiver impedance ${\hat{Z}}_{r}$ is real, leading to real defect impedances ${\hat{Z}}_{d_{i}}$, ${\hat{Z}}_{d_{i+1}}$ and ${\hat{Z}}_{d_{i,i+1}}$. However, the equivalent impedance ${\hat{Z}}_{eq}\left(x\right)$ is real only in the case of perfect alignment of the receiver with an array resonator. In general, it presents complex values, as it is possible to see from the blue and red curves in Figure 7, Figure 8 and Figure 9. This is due to the presence of the term ${\hat{D}}_{i,i+1}$, which is always complex and becomes null only if the receiver couples with one array resonator at a time (since real defect impedances are now considered). Intuitively, this behaviour indicates that the receiver introduces a further coupling between the resonators it faces, with a consequent phase delay in the equivalent impedance.

#### 3.2.2. Non-Resonant Receiver

For complex values of ${\hat{Z}}_{r}$, the behaviour of ${\hat{Z}}_{eq}\left(x\right)$ slightly changes. In particular, $|{\hat{Z}}_{eq}\left(x\right)|$ shows maxima and minima in correspondence of perfectly alignment positions, similarly to the case of the perfectly resonant receiver. The phase $\varphi_{{\hat{Z}}_{eq}}\left(x\right)$ is shifted with respect to the previous case and, with the reactive loads considered here, it presents extreme values for perfect alignment positions and null values when the receiver is between two array resonators, for which the equivalent impedance is then real. From the yellow and violet curves of Figure 7, Figure 8 and Figure 9, it can also be noticed that the trend of ${\hat{Z}}_{eq}\left(x\right)$ for inductive and capacitive receiver impedances is opposite, as expected. Overall, for increasing values of $|{\hat{Z}}_{r}|$, the defect impedances (12) are smaller, and the phase shift of ${\hat{Z}}_{eq}\left(x\right)$ is less pronounced.

### 3.3. Termination Conditions and Function Univocity

**Figure 10:**
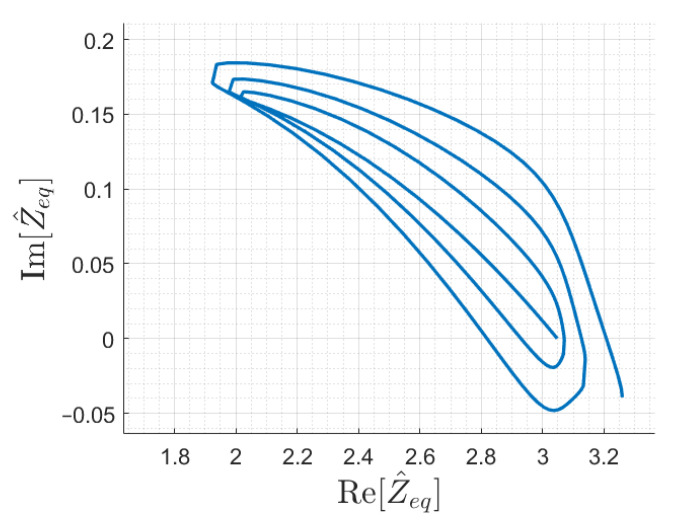
Input impedance trajectory as a function of the receiver position for the resonator array with a series-compensated receiver and short-circuit termination.

Another peculiarity of the input impedance function is its one-to-one behaviour with respect to the receiver position *x*. Indeed, even if both magnitude and phase of ${\hat{Z}}_{eq}$ are periodic with respect to the receiver position, their combination can be unique for any *x*. The fulfillment of this requirements can be verified studying the trajectory of ${\hat{Z}}_{eq}$ in the complex plane according to the receiver position *x* for certain values of ${\hat{Z}}_{T}$, which should not present intersection points. It is plotted in Figure 10 considering the inductive receiver load ${\hat{Z}}_{r}$ of the test case and ${\hat{Z}}_{T}=0$. The plot shows that intersections might occur, and, in that case, the value of ${\hat{Z}}_{eq}$ would correspond to different *x* positions.

Typical termination conditions for resonator arrays discussed in literature are the perfect matching, open- and short- circuits [30,33], which correspond to ${\hat{Z}}_{T}\approx \omega M$, ${\hat{Z}}_{T}\to \infty$ and ${\hat{Z}}_{T}=0$, respectively.

**Figure 11:**
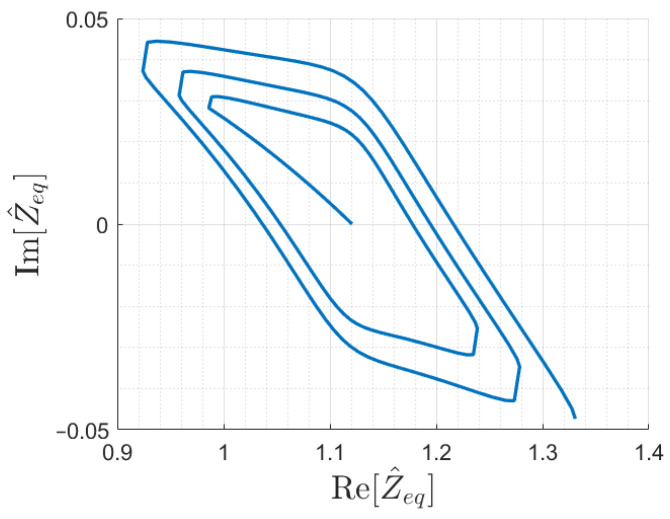
Input impedance trajectory as a function of the receiver position for the resonator array with six cells and series-compensated receiver, terminated with ${\hat{Z}}_{T1}=0.8\;\Omega$.

**Figure 12:**
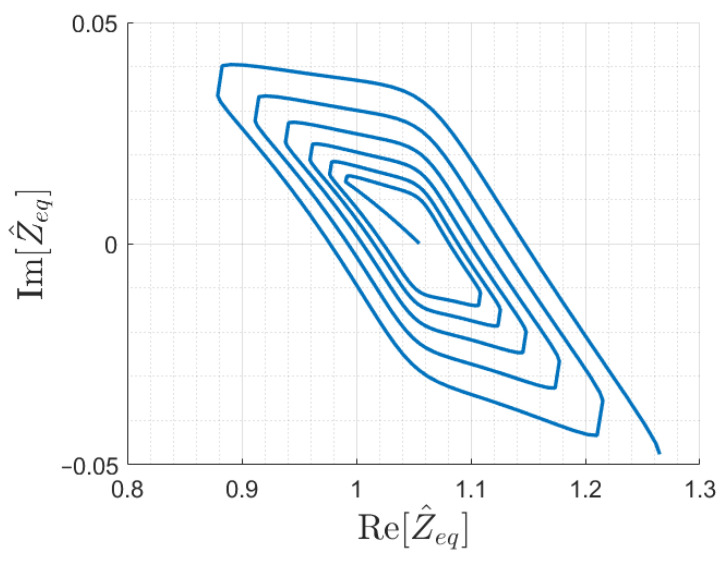
Input impedance trajectory as a function of the receiver position for the resonator array with twelve cells series-compensated receiver, terminated with ${\hat{Z}}_{T1}=0.8$.

However, to ensure univocity, simulations proved the need of introducing different array terminations. In particular, a suitable termination impedance has been found for the six-cell array considered so far, with a value of ${\hat{Z}}_{Tl}\approx 0.8\;\Omega$. The input impedance trajectory for an array terminated with ${\hat{Z}}_{Tl}$ is shown in Figure 11. The correct behavior has been verified also for a longer array of 12 cells, and the resulting ${\hat{Z}}_{eq}$ is shown in Figure 12.

## 4. Outline of the Algorithm for the Moving-Coil Position Detection

The estimation of the receiver coil position *x* can be carried out in different manners. The basic idea consists of feeding the first array resonator with a sinusoidal voltage $v_{1}\left(t\right)$ at the resonant frequency $f_{1}$ and measure the current circulating in the same resonator $i_{1}\left(t\right)$, from which the input impedance can be estimated for any position of the receiver as:

(15) $$\tilde{Z}=\frac{|{\hat{V}}_{1}|}{|{\hat{I}}_{1}|}$$

where ${\hat{I}}_{1}$ and voltage ${\hat{V}}_{1}$ are the phasors of the input current and voltage at $\omega_{1}$, respectively. The superscript “∼” denotes estimated quantities.

Then, using the estimated and (theoretically) calculated impedance values, it is possible to determine the occupied coil by evaluating the change of sign of impedance differential (basic algorithm), or more accurately estimate the receiver position *x* for each discretization step Δx.

### 4.1. Algorithm

Based on the analysis presented in Section 3.2 and practical considerations, in a DWPT system, the following assumptions can be made:

- the function $|{\hat{Z}}_{eq}\left(x\right)|$ is monotonic in the interval between two positions of perfect alignment;
- the receiver couples consecutively with all the array coils starting from the first one.

The coils *i* and (i+1) directly coupled with the receiver can be identified considering the derivative of the input impedance magnitude and the second assumption. Indeed, each time the receiver couples with a new array coil, the slope of ${\hat{Z}}_{i,i+1}^{eq}\left(\xi \right)$ reverses. Thus, when the vehicle enters the array, the receiver initially couples to the first array coil, namely i=1, and then the position *i* and i+1 can be updated each time a change in the $d_{|\hat{Z}|}$ sign occurs. The check of the function derivative can be easily done evaluating the difference between function values at consecutive positions as:

(16) $$\Delta {\tilde{Z}}_{k}\;=\;{\tilde{Z}}_{k+1}-{\tilde{Z}}_{k}$$

where ${\tilde{Z}}_{k}$ and ${\tilde{Z}}_{k+1}$ are the estimated impedance values associated with two successive positions $x_{k}$ and $x_{k+1}$, respectively. It is underlined that the knowledge of such two positions is not necessary to detect the change of sign of the calculated differential.

Assuming monotonicity as stated above and knowing the array coils *i* and (i+1) coupled with the receiver, the function $|{\hat{Z}}_{eq}\left(x\right)|$ is sufficient to estimate the receiver position *x*. In fact, it is possible to limit the search of the receiver position to the correct coil interval, where the impedance curve is locally monotonic. Equivalently, this implies removing problems due to poor separation at some points of the oblique parts of the impedance trajectories, shown in Figure 10, Figure 11 and Figure 12.

Once the coupled resonators *i* and i+1 are found, the position *x* can be determined through (6) and enforcing:

(17) $$\|\tilde{Z}-|{\hat{Z}}_{i,i+1}^{eq}\left(\xi \right)\left|\right\|\leq \delta_{Z}$$

where $\delta_{Z}$ is the accepted tolerance margin.

To further speed up the position detection process, it is possible to update the initial reference position with the one found in the step before.

Finally, it can be noticed that, in case the algorithm is used to decide which coil must be turned on (i.e., the exact receiver position is not required), the knowledge of which coils *i* and i+1 the receiver is coupled to is sufficient. The basic algorithm is visible embedded in the pseudo-code shown in Algorithm 1 (see comment “change of coil”).
**Algorithm 1:** Receiver coil position detection${\tilde{Z}}_{0}=0$k=1i=1**while**x<nd**do**      Acquire ${\tilde{Z}}_{k}$      **if** $sgn\left[\Delta {\tilde{Z}}_{k}\right]\neq sgn\left[\Delta {\tilde{Z}}_{k-1}\right]$ **then**            i=i+1                       ▷ Change of coil      **end if**      ξ=0      **while** $\|{\tilde{Z}}_{k}-|{\hat{Z}}_{i,i+1}^{eq}\left(\xi \right)\left|\right\|>\delta_{Z}$ **do**            ξ←ξ+Δx      **end while**      $x_{k}=x_{k-1}+\xi$      k=k+1**end while**                           ▷ End of array

### 4.2. Sensitivity

The number of array coils and the sensitivity of the input impedance function to the coordinate *x* determine the spatial resolution. The sensitivity of ${\hat{Z}}_{eq}\left(x\right)$ is crucial to determine the effective accuracy and can be defined for both its amplitude and phase as, respectively,

(18) $$s_{mag}\left(x\right)=\frac{d|{\hat{Z}}_{eq}\left(x\right)|}{dx}$$

and

(19) $$s_{ph}\left(x\right)=\frac{d\varphi_{{\hat{Z}}_{eq}}\left(x\right)}{dx},$$

Theoretically, for an accurate measurement, they should present the largest magnitude possible, even though they risk diverging for some *x*, making their calculation very difficult. Practically, the sensitivity is mainly affected by the space variation of the mutual inductance $M_{r,i}\left(x\right)$, which contributes to ${\hat{Z}}_{eq}\left(x\right)$, as shown in (10) and (12). Indeed, whereas the number of positive and negative peaks of both $|{\hat{Z}}_{eq}\left(x\right)|$ and $\varphi_{{\hat{Z}}_{eq}}\left(x\right)$ depends on the number of resonators, their variation for a receiver coupled with the generic *i*th and (i+1)th cells depends on $dM_{r,i}\left(x\right)/dx$.

On the other hand, a higher number of array resonators leads to an input impedance function more sensitive to position *x*, since it presents more periods and thus a more pronounced variation in space. As a consequence, it also defines the smaller discretization step Δx, while the larger is defined based on the maximum accepted measurement error and the microprocessor which performs the calculations.

### 4.3. Outline of Hardware Implementation and Computational Effort

The purpose of this subsection is to demonstrate the feasibility of the hardware implementation for what regards the measurement of the physical quantities, their processing and the execution of the detection algorithm.

As anticipated in Figure 4, the impedance estimate is based on the $v_{1}\left(t\right)$ and $i_{1}\left(t\right)$ waveforms measurement and extraction of the respective phasors at the test frequency $f_{1}$. The voltage measurement can be carried out by using a compensated voltage divider, galvanically isolated by a cascaded optically isolated voltage transducer. The current measurement can be carried out similarly reading the current with a resistive shunt optically buffered by the same device. These optically isolated voltage transducers (such as AMC1202 [34], AMC3301 [35] and ACPL-790 [36]) have a bandwidth of 200 kHz or larger, an input noise density of less than 1 μV, and a basic forward gain of 8.2 (41 for reduced input scale of ± 50 mV). Gain error and drift are below about ±0.2% and ±0.1%, accounting for various sources of variability, including a temperature change of more than 30 °C; the impact on impedance uncertainty is thus limited to less than ±0.3%, using rms composition of errors of similar voltage and current readings. The input noise as a source of uncertainty is irrelevant, as the estimate of voltage and current phasors will be always done with a narrow enough bandwidth Δf of at most some hundreds Hz centered round $f_{1}$. In this case, the overall rms noise is about 10 μV and for signals in excess of 10 mV, as it is the case for the selected isolated amplifiers, and it represents a contribution of less than 0.1%.

This demonstrates the feasibility of the hardware implementation of the impedance measurement. Computational complexity and real-time implementation are considered in the following.

Sampling can be carried out at 1 MSa/s. There is no necessity of performing a full FFT, except for initialization; then, the $f_{1}$ components $V(f_{1})$ and $I(f_{1})$ are estimated with a recursive FFT together with the adjacent components at $f_{1}-\Delta f$ and $f_{1}+\Delta f$, to track stability of $f_{1}$. The complexity would be limited to a few floating point operations. With Δf=300 Hz, the number of samples is $N=f_{s}/\Delta f=333$, that is, in any case, a manageable size for a full FFT: with a FFT complexity $O\left(N\right)=2N\log_{2}\left(N\right)$, it would require 5600 floating point operations. Modern DSPs have computational power in excess of several hundred MFlops, floating point operations per second (exemplified by [37,38] covering almost 20 years of DSP production). The two FFTs for $V_{1}\left(f_{1}\right)$ and $I_{1}\left(f_{1}\right)$ plus other minor computations would be carried out in less than 120 μs.

The proposed algorithms for estimation of the vehicle position and of the occupied cell also necessitate a limited number of operations. Provided that sampling of two quantities (voltage and current at the measuring port) is carried out independent of the CPU by means of DMA (direct memory access), remaining calculations are the estimate of the voltage and current phasors, their ratio to obtain the impedance and then the implementation of the two algorithms for the necessary number of steps. Both algorithms can run every time starting from the previously calculated vehicle position or active coil number, so that the number of iterations is limited to one or two.

The impedance at each algorithm run is calculated with one complex operation. The initialization of the loop and comparison of measured and calculated impedance values with the absolute value operation consist of about 4 + 6 operations plus some conditional jumps—similarly to the algorithm for occupied coil detection that implements a derivative. Complex operations (including addition, multiplication, division, square and square root) may be assumed to have all of the same complexity for simplicity, although the latter two always require some more CPU cycles. It is easy to see that the total number of floating point operations is limited to some hundreds with margin, adding nothing relevant to the already calculated 120 μs.

The attainable spatial resolution of the complete set of algorithms with a vehicle traveling at 100 km/h would then be much smaller than the already satisfactory 2.8 cm resolution at a conservative 1 ms cycle time.

## 5. Conclusions

A new passive sensing technique that is applicable to a longitudinal array structure of magnetically coupled resonators has been presented and discussed: the position of a receiver coil moving over the array is estimated by measuring the array input impedance. The method is suitable for various types of WPT architectures, having considered in the present work mainly an IPT for electric vehicle applications.

The sensing method has been demonstrated by solving recursively the array equivalent circuit including the effect of the receiver coil at a variable position. The peculiar behavior of the input impedance curve shown in Section 3.2 allows the unambiguous determination of the receiver coil position. The analytical model has been tested considering a real case scenario, where the system parameters have been chosen according to the current standard for static WPT systems, as a benchmark. With the proper termination impedance, the sensing of the receiver coil is demonstrated. As a future work, optimized values of the termination impedance can be investigated in more detail to further improve the performance of the proposed sensing method, increasing its accuracy.

The method could be in principle extended to various IPT architectures and such demonstration should be supported by an extensive verification of existing solutions that at the moment are not standardized yet. A practical implementation is a foreseeable advancement of the present work, including realistic parasitic terms and parametric changes, in addition to accounting for the effect of the feeding inverter.

## Figures and Tables

**Table 1:** Resonator Array parameters.

Quantity	Symbol	Value
Quality factor	*Q*	300
Primary Coils Self-inductance	*L*	70 μH
Primary Coils Mutual Inductance	*M*	−2.6 μH
LCC Series Capacitance	*C*	82.3 nF
LCC Series Inductance	$L_{f}$	27.34 μH
LCC Parallel Capacitance	$C_{f}$	0.128 μF
WPT Resonance Frequency	$f_{0}$	85 kHz
Z measurement Frequency	$f_{1}$	108.365 kHz
Array Matching Impedance	${\hat{Z}}_{match}$	1.82 Ω

**Table 2:** Receiver parameters.

Quantity	Symbol	Value
Receiver Coil Quality factor	$Q_{r}$	300
Receiver Coil Self-inductance	$L_{r}$	40 μH
Receiver Series Capacitance	$C_{r}$	87.5 nF
WPT Resonance Frequency	$f_{0}$	85 kHz
On-board DC-bus Voltage	$V_{{OBC}_{dc}}$	520 V
Power Rate	*P*	520 kW

## Data Availability

Data are available in the paper.
